# CARE DEPENDENCY IN OLD AGE IN MEXICO: SITUATIONAL ANALYSIS, IMPLICATIONS, AND RECOMMENDATIONS

**DOI:** 10.1093/geroni/igz038.250

**Published:** 2019-11-08

**Authors:** Mariana López-Ortega, Luis Miguel F Gutiérrez Robledo

**Affiliations:** 1 National Institute of Geriatrics, Mexico City, Mexico; 2 National Institute of Geriatrics, Mexico City, Distrito Federal, Mexico

## Abstract

The demographic transition, population aging, and the epidemiological transition in Mexico have modified the burden of the disease profile, and impose important economic, social, and health care challenges, particularly old-age and disability-related care dependency. We describe old age health and dependency conditions, and analyze the supply of services to dependent population, including aging and disability norms and policies, the role of the government and the private sector (profit & non-profit) in the provision of related services. Based on the analysis, it is clear that public strategies for care dependency are practically non-existent and that policies aimed at these populations focus on guaranteeing access to basic health services, an economic income, and social integration. Also, there are no support strategies for family caregivers who currently carry most of the care work. Care dependency strategies to support those who need them as well as those who currently provide them are urgently needed.

